# Feasibility and Acceptability of a Smoking Cessation Smartphone App (My QuitBuddy) in Older Persons: Pilot Randomized Controlled Trial

**DOI:** 10.2196/24976

**Published:** 2021-04-14

**Authors:** Jenny Peek, Karen Hay, Pauline Hughes, Adrienne Kostellar, Subodh Kumar, Zaheerodin Bhikoo, John Serginson, Henry M Marshall

**Affiliations:** 1 The University of Queensland Thoracic Research Centre The Prince Charles Hospital Chermside Australia; 2 QIMR Berghoffer Medical Research Institute Brisbane Australia; 3 The Department of Respiratory Medicine Redcliffe Hospital Redcliffe Australia; 4 The Pharmacy Department The Royal Brisbane and Women's Hospital Brisbane Australia; 5 The Department of Respiratory Medicine Caboolture Hospital Caboolture Australia

**Keywords:** mHealth, mobile apps, motivation, smartphone, smoking cessation, tobacco smoking

## Abstract

**Background:**

Although many smoking cessation smartphone apps exist, few have been independently evaluated, particularly in older populations. In 2017, of the 112 commercially available smoking cessation apps in Australia, only 6 were deemed to be of high quality, in that they partially adhered to Australian guidelines. Mobile health (mHealth) apps have the potential to modify smoking behavior at a relatively low cost; however, their acceptability in older smokers remains unknown. Rigorous scientific evaluation of apps is thus urgently needed to assist smokers and clinicians alike.

**Objective:**

We conducted a pilot randomized controlled trial to evaluate the feasibility of a large-scale trial to assess the use and acceptability of a high-quality smoking cessation app in older smokers.

**Methods:**

Adult inpatient and outpatient smokers with computer and smartphone access were recruited face to face and via telephone interviews from Metropolitan Hospitals in Brisbane, Australia. Participants were randomized 1:1 to the intervention (requested to download the “My QuitBuddy” smoking cessation app on their smartphone) or the control group (provided access to a tailored smoking cessation support webpage [Quit HQ]). The My QuitBuddy app is freely available from app stores and provides personalized evidenced-based smoking cessation support. Quit HQ offers regular email support over 12 weeks. No training or instructions on the use of these e-resources were given to participants. Outcomes at 3 months included recruitment and retention rates, use and acceptability of e-resource (User Version of the Mobile App Rating Scale [uMARS]), changes in quitting motivation (10-point scale), and self-reported smoking abstinence.

**Results:**

We randomized 64 of 231 potentially eligible individuals (27.7%). The mean age of participants was 62 (SD 8). Nicotine dependence was moderate (mean Heaviness of Smoking Index [HSI] 2.8 [SD 1.2]). At 3 months the retention rate was (58/64, 91%). A total of 15 of 31 participants in the intervention arm (48%) used the app at least once, compared with 10 of 33 (30%) in the control arm. uMARS scores for e-resource use and acceptability were statistically similar (*P*=.29). Motivation to quit was significantly higher in the intervention arm compared with the control arm (median 6 [IQR 4-8] versus 4 [IQR 4-5], respectively, *P*=.02). According to the intention-to-treat analysis, smoking abstinence was nonsignificantly higher in the intervention group (4/31 [13%], 95% CI 4%-30%, versus 2/33 [6%], 95% CI 1%-20%; *P*=.42). The estimated number needed to treat was 14.

**Conclusions:**

Internet and mHealth smoking cessation resources appear acceptable to a minority of older smokers. Smokers who engaged with the allocated e-resources rated them equally, and there were trends toward greater uptake, increased motivation, and higher abstinence rates in the app group; however, only the change in motivation reached statistical significance (median score 6 versus 4, respectively, *P*=.02). This results of this pilot study suggest that apps may improve quit outcomes in older adults who are willing to use them. Further research into user–app interactions should be undertaken to facilitate improvements in app design and consumer engagement. These favorable trends should be explored in larger trials with sufficient statistical power.

**Trial Registration:**

Australian New Zealand Clinical Trials Registry ACTRN12619000159156; http://www.anzctr.org.au/Trial/Registration/TrialReview.aspx?id=376849&isReview=true

## Introduction

Globally, 6 million people die from tobacco use annually, accounting for 11.5% of deaths worldwide and an economic cost of US $1 trillion [1]. Despite smoking prevalence falling in most economically developed countries [2], important efforts to reduce prevalence are ongoing [3]. With an estimated 6.8 billion active mobile phones worldwide, patient-facing smartphone apps offer novel opportunities to modify health behavior at low cost. With little or no clinician input they may represent a powerful new platform to help smokers quit [4,5]. Multiple smoking cessation apps exist; however, only few have been independently evaluated in clinical populations. Thornton et al [6] reviewed all free, commercially available smoking cessation apps in Australia, and only 6 of 112 apps were deemed high quality, at least partially following Australian treatment guidelines. Haskins et al’s [7] systematic review identified only 6 smoking cessation apps with peer-reviewed scientific support. Only 2 (4%) of the top 50 suggested by leading app stores had any scientific support. In addition, most app trials have been small, resulting in imprecise effect estimates. A meta-analysis of 8 randomized controlled trials (n=3543) found a clinically, but nonstatistically significant change in the rate of abstinence compared with usual care (pooled relative risk 1.15, 95% CI 0.85-1.57) [8].

No trials so far have assessed older populations; mean age ranges in published trials vary from 24.9 to 54.3 years [8]. Older populations are important as they represent heavier tobacco users and perhaps harder-to-treat smokers who may be less fluent in, or have limited access to, information technology (IT) [9,10]. Thus, there is an urgent need for data in this cluttered and poorly regulated market across all population subgroups, especially in older smokers.

My QuitBuddy, one of the most popular apps with over 200,000 downloads [11], was released in 2012 by the Australian Department of Health and is in the top 10 recommended smoking cessation apps in iOS and Google Play stores. Although deemed high quality, it lacks evaluation in a randomized controlled trial [6].

We therefore designed the eQUIT study to assess the feasibility, use, and acceptability of the My QuitBuddy smoking cessation app in adult smokers. A secondary outcome was to estimate the treatment effect size to inform power calculations for larger trials.

## Methods

### Study Design and Participants

eQUIT was a randomized controlled trial of smoking cessation e-resources. Current smokers aged over 18 who owned an internet-enabled smartphone and a computer were eligible. Smokers currently using a smoking cessation app were ineligible. Recruitment was limited to 1 participant per household. Control arm participants were instructed to not download any smoking cessation apps during the study period. Screening and mixed methods recruitment (face to face/telephone) were via smoking cessation clinics and inpatient and outpatient respiratory clinics (Multimedia Appendix 1). We also included enrollees in the International Lung Screen Trial (ILST) [12] in the Metro North Hospital and Health Service, Brisbane. Potentially eligible adult smokers were identified from clinics by the research team and from the ILST study database. Potentially eligible participants were asked questions to determine their eligibility (smartphone and computer ownership, concurrent app use). If eligible, participants were invited to provide informed consent which they could accept or decline. While recruitment was open to all adults, we primarily targeted older smokers, as clinical patients with chronic disease seen in hospital respiratory and cardiac clinics tend to be older; additionally, all participants undergoing lung cancer screening in the ILST were aged between 55 and 80 (an eligibility criterion of the ILST).

The trial was registered with the Australian New Zealand Clinical Trials Registry (ACTRN12619000159156) and approved by the hospital human research ethics committees.

### Smoking Cessation e-Resources

Participants in the control arm received an access link to a smoking cessation webpage hosted by the Queensland State Department of Health (Quit HQ) [13]. Quit HQ allows registration for a 12-week program of support emails containing health advice, motivational stories, and Quitline links.

**Figure 1:**
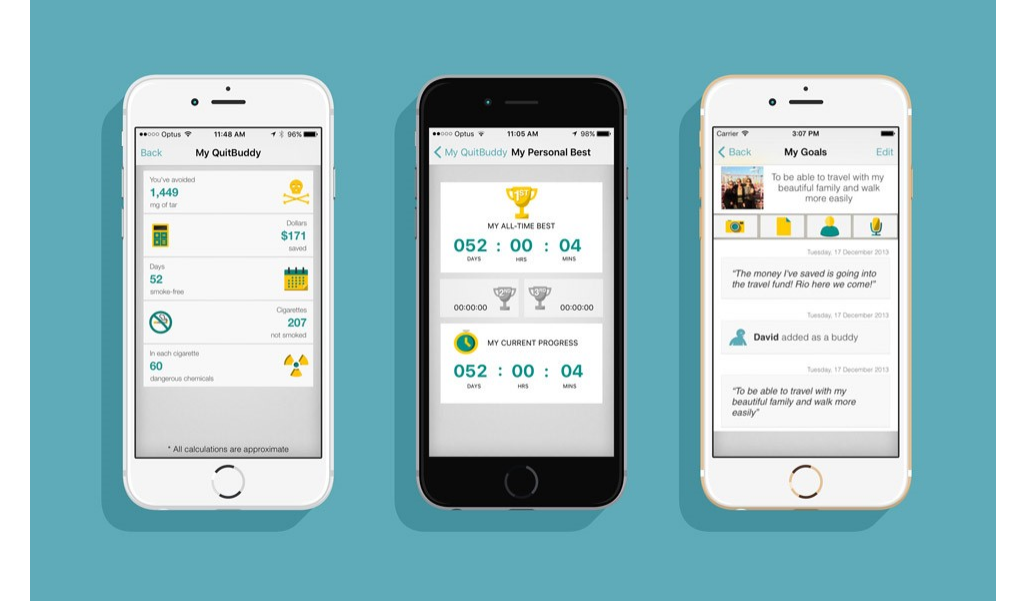
Screenshot My QuitBuddy app.

Participants in the intervention arm received the link to download the My QuitBuddy app from app stores (Figure 1) [11]. My QuitBuddy motivates users across 4 functional domains: rational (health benefits, cost savings); emotional (positive influence of family and friends); social (community forums); and gamification (playful interactions producing serious outcomes). Both e-resources utilize similar educational content, motivational techniques, and direct links to Quitline. However, the app provides more personalized support in real time.

### Randomization

Randomization codes, stratified by hospital and grouped in blocks of 6, were centrally generated by a computer [14]. Allocation was concealed using sequentially numbered opaque envelopes.

Participants were randomized 1:1 after written consent and baseline questionnaires were completed. Participants were provided with a download link to the respective e-resource (thus not blinded). No training in e-resource use was given to simulate “real life” more closely. Participants were instructed to engage with the e-resource at their own discretion and use any standard-of-care smoking cessation interventions provided by their primary care provider.

### Data Collection

Participants completed questionnaires at baseline and 3 months after randomization. Questionnaires were collected by telephone, in person, post, or email.

Baseline data included demographics; smoking history; smoking-related disease; motivation to quit (10-point scale, ranging from 1 [I enjoy smoking so much I will never consider quitting] to 10 [I have quit and I am 100% confident that I will never smoke again]) [15]; and generic health-related quality of life [16] (5-level EQ-5D [EQ-5D-5L], with higher scores indicating worse health status, EQ-5D Visual Analogue Scale [EQ-VAS] score [16], and self-reported overall health, ranging from 100 [“best possible” health] to 0 [“worst possible” health]).

IT proficiency was rated by frequency of simple tasks (internet shopping and banking) and self-rated IT confidence (visual analog scale 0-100, with higher scores indicating higher confidence). Nicotine dependence was assessed using the HSI [17].

Outcome assessment was unblinded; however, subjective assessment was minimized by using a standard questionnaire. Self‐reported smoking abstinence at 3 months was defined as smoking less than 5 cigarettes in the previous 12 weeks [18]. Visits to general practitioner and cessation intervention received (5As; “Ask, Advise, Assess, Assist, and Arrange”) [19] were recorded. Participants rated the e-resource using the User Version of the Mobile App Rating Scale (uMARS), a 20-item measure with 4 objective quality subscales (engagement, functionality, aesthetics, and information quality); a subjective quality subscale; and a perceived impact subscale. Higher scores indicate better quality rating [20]. As far as we are aware, there are no validated tools that can be used across every type of internet or app resource. As we wanted to compare responses using the same tool across both conditions, we chose uMARS because (1) uMARS questions appear equally relevant to webpages as they do for apps, thus the same questionnaire could be administered to all participants; (2) the uMARS tool did not require substantial alteration for website users (wherever the term “app” appeared in the tool, we changed the wording to “app/webpage”; the questions themselves did not require any adjustment); (3) uMARS has been very well validated in many settings and the focus of this study was to evaluate an app as the experimental condition, rather than webpage as the control condition.

The scheduled in-app and email reminders of both the intervention and control e-resources were individualized by the participants if and when they first engaged with their allocation. This information was not collected by the research team. Up to 10 attempts to contact participants to complete questionnaires were allowed during follow-up as per protocol.

### Sample Size Justification and Statistical Analysis

As a feasibility study, formal sample size calculation was not required. However, we estimated the sample size required at around 10% of the number required for a statistically powered study. Assuming less than 20% loss to follow-up, a sample size of 64 would inform our aims of determining feasibility and acceptability [21,22].

Intention-to-treat analysis was performed, assuming nonresponders were smokers. Continuous measures were summarized using mean or median and compared using Student *t* test (for normally distributed data) or Wilcoxon rank-sum test (if data are not normally distributed). Categorical variables were compared using the Fisher exact test. Analyses were performed using the Stata Software (version 15; StataCorp). *P* values less than .05 were considered statistically significant.

### Data Sharing Statement

Anonymized data that support the findings of this study are available on request from the corresponding author, subject to ethical review. The data are not publicly available due to privacy and ethical restrictions.

## Results

### Recruitment

**Figure 2:**
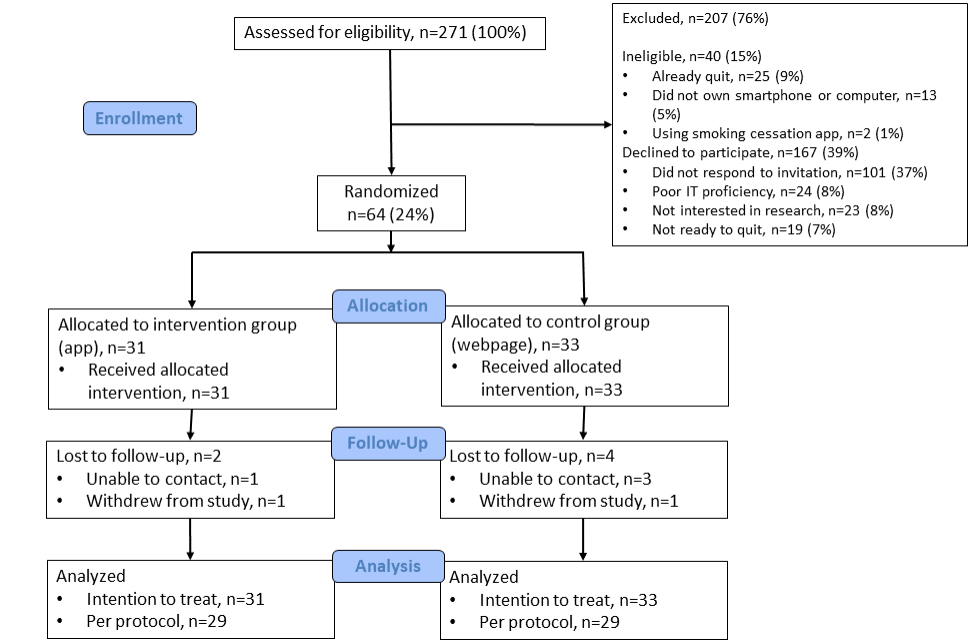
eQUIT consort.

Between the April 4 and May 23, 2019, 271 inpatient and outpatient smokers were screened for eligibility (Figure 2). Of the 231 potentially eligible individuals, 64 (27.7%) were randomized. A total of 31 were allocated to the intervention arm and 33 to the control arm. Reasons for non-enrollment are outlined in Figure 2.

### Baseline Data

Baseline characteristics are presented in Table 1. Overall, 29/64 (45%) participants were female, and 34/64 (53%) participants had completed tertiary-level education. The mean age was 62 (SD 8), and only 4 participants were under the age of 50. Participants smoked a median of 15 cigarettes per day. Average nicotine addiction was moderate and similar between groups (mean HSI was 2.5 and 3.0, *P*=.18). Median motivation to quit was 4, equating to the statement “I sometimes think about quitting but have no specific plans to quit.” Nearly all participants (60/64, 94%) reported previous quit attempts. Only the history of chronic obstructive pulmonary disease was statistically significantly higher in the control arm (9/33 [27%] vs 1/31 [3%], *P*=.013).

Health-related quality of life was good. Proficiency in IT tasks appeared quite high; over one-half of the group used internet banking every week, and over one-half used internet shopping regularly. Self-rated IT confidence was high (median 75 out of 100).

**Table 1:** Baseline demographic data.

Demographics			Intervention arm (My QuitBuddy app; n=31)	Control arm (Quit HQ Webpage; n=33)	*P* value
Age (years), mean (SD)			61 (9)	63 (8)	.52^a^
Gender (female), n (%)			11 (35)	18 (55)	.14^b^
**Education, n (%)**					.10^b^
	Grade 12 or less		13 (42)	11 (33)	
	Completed high school		5 (16)	1 (3)	
	Tertiary/vocational/post graduate level achieved		13 (42)	21 (64)	
History of asthma, n (%)			4 (13)	9 (27)	.22^b^
History of chronic obstructive pulmonary disease, n (%)			1 (3)	9 (27)	.013^b^
History of bronchitis, n (%)			0 (0)	2 (6)	.49^b^
History of emphysema, n (%)			9 (29)	11 (33)	.79^b^
History of heart disease, n (%)			3 (10)	5 (15)	.71^b^
History of cancer, n (%)			3 (10)	5 (15)	.71^b^
Heaviness of Smoking Index^c^, mean (SD)			2.5 (1.5)	3 (1.0)	.18^a^
Cigarettes per day, median (IQR)			20 (10-20)	15 (10-20)	.74^d^
At least one prior quit attempt, n (%)			29 (94)	31 (94)	>.99^b^
Motivation to quit, median (IQR)			4 (4-6)	4 (4-5)	.86^d^
**Health-related quality of life^e^, median (IQR)**					
		Anxiety/depression	0 (0-1)	0 (0-1)	.63^d^
		Mobility	0 (0-1)	0 (0-1)	.50^d^
		Pain/discomfort	1 (0-1)	0 (0-1)	.20^d^
		Self-care	0 (0-0)	0 (0-0)	.65^d^
		Usual activities	0 (0-1)	0 (0-0)	.07^d^
		EQ-5D Visual Analogue Scale score	70 (50-85)	80 (70-90)	.14^d^
**Online banking frequency, n (%)**					.84^b^
		Never	7 (23)	6 (18)	
		Occasional to regular use	7 (23)	10 (30)	
		Every week	17 (55)	17 (52)	
**Online shopping frequency, n (%)**					.59^b^
		Never	13 (42)	12 (36)	
		Occasional to regular use	18 (58)	19 (58)	
		Every week	0 (0)	2 (6)	
Information technology confidence, median (IQR)			80 (60-85)	70 (50-90)	.77^d^

^a^From independent *t* test.

^b^From Fisher exact test.

^c^Higher score indicates greater addiction.

^d^Wilcoxon rank-sum test.

^e^Measured using the EuroQol-5D instrument.

### Outcomes

Of the 64 participants randomized, 58 (91%) completed a follow-up questionnaire at 12 weeks. An equal number of participants in each arm completed the study per protocol (29/31 [94%] and 29/33 [88%]), respectively.

e-Resource engagement was higher in the intervention arm: 15/31 (48%; 95% CI 32%-70%) participants in the intervention arm used the resource (app) at least once, compared with 10/33 (30%; 95% CI 18%-54%) participants in the control arm (*P*=.29). uMARS ratings of e-resource across 7 subscales did not differ significantly between intervention and control arms, with weak evidence for a higher functionality rating for the app (*P*=.07 to *P*=.51). Both e-resources had a median star rating of 3 out of 5 stars (Table 2).

In total, 42 participants (from both intervention and control arms) had visited their primary care provider at follow-up, and they equally utilized pharmacotherapy (Table 2). In both arms, provision of the more “active” parts of the 5As intervention was similar (“assist” and “arrange”).

**Table 2:** Distribution of responses at follow-up by the study arm.

Response			Intervention arm (My QuitBuddy app; n=29)		Control arm (Quit HQ Webpage; n=29)		*P* value
Smoking abstinence, n (%)			4 (14)		2 (7)		.67^a^
Heaviness of smoking index, mean (SD)			2.0 (1.5)		2.2 (1.3)		.50^b^
Motivation to quit, median (IQR)			6 (4.0-8.0)		4 (4.0-5.0)		.02^c^
Cigarettes per day, median (IQR)			10 (2-20)		12 (6-20)		.31^c^
Ever-use of e-resource, mean (SD)			15 (52)		10 (34)		.29^a^
e-Resource star rating, median (IQR)			3 (3-4)		3 (3-4)		.24^c^
**User Version of the Mobile App Rating Scale, mean (SD)**							
	Engage	2.7 (0.9)		2.4 (1.0)		.50^b^	
	Function	3.7 (0.9)		2.9 (1.3)		.07^b^	
	Aesthetics	3.5 (0.7)		3.1 (0.9)		.17^b^	
	Information	3.9 (0.8)		3.7 (1.2)		.51^b^	
	Quality	3.3 (0.9)		3.0 (1.1)		.43^b^	
	Subjective quality	2.9 (1.2)		2.3 (1.2)		.15^b^	
	Impact	3.4 (1.1)		2.9 (1.4)		.33^b^	
**Pharmacotherapy, n (%)**							
	Varenicline	5 (17)		5 (17)		>.99^b^	
	Bupropion	1 (3)		0 (0)		>.99^b^	
	Nicotine patches	6 (21)		7 (24)		>.99^b^	
**General practitioner follow-up, n (%)**							
	Visited general practitioner	22 (76)		20 (69)		.77^a^	
	Ask/advise/assess	11^d^ (50)		14^e^ (70)			
	Assist/arrange medication counselling/follow-up	9^d^ (41)		8^e^ (40)			

^a^From Fisher exact test.

^b^From independent *t* test.

^c^Wilcoxon rank-sum test.

^d^N=22.

^e^N=20.

Quit motivation was significantly higher in the intervention arm than in the control arm (median score 6 versus 4, respectively, *P*=.02), equating to the following statements: “I plan to quit in the next 6 months” and “I sometimes think about quitting but have no specific plans to quit,” respectively. Mean cigarettes smoked per day decreased by half in the intervention arm, although this was not statistically significant (*P*=.31).

Using per-protocol analysis, 4/29 (14%; 95% CI 4%-32%) participants in the intervention arm and 2/29 (7%; 95% CI 1%-23%) in the control arm reported quitting at 3 months (*P*=.67). In the intention-to-treat analysis, the respective proportions were 4/31 (13%; 95% CI 4%-30%) and 2/33 (6%; 95% CI 1%-20%). The relative risk of quitting smoking in the intervention arm was 2.1 (95% CI 0.4-10.8; *P*=.42). The number of participants needed to treat for 1 successful quitter was 14 [23]. The number needed to treat was calculated as the inverse of the absolute risk reduction.

## Discussion

### Principal Findings

We conducted a pilot randomized controlled trial to assess the uptake, use, and acceptability of the My QuitBuddy app in an adult population compared with a webpage presenting resources for quitting smoking. The app appeared reasonably acceptable to smokers. Although both e-resources received similar ratings for engagement, functionality, aesthetics, and information quality, we found a trend toward greater uptake of the app, which may reflect greater convenience and immediacy of a smartphone platform. While participants in both arms appeared equally well supported by their primary care physicians, we observed a clinically and statistically significant increase in motivation to quit among users of the app at 3 months, and a nonstatistically significant, but clinically very significant, halving of mean daily cigarette consumption and doubling in self-reported quit rate. These results suggest tangible benefits for smokers using the app. Although this pilot trial was underpowered to detect small differences in outcomes, the observed trends are encouraging and worth pursuing in a larger trial. Even if the absolute difference in quit rate is small, potential benefits at the population level could be enormous, considering the reach of smartphones. For example, Phase 1 of the Australian National Tobacco Media Campaign in 1997 reduced national smoking prevalence by 1.4%, resulting in health care savings of AUD 740.6 (US $572.08) million [24].

### Digital Literacy and Access

Digital technology to help smokers is not a panacea and certainly not a replacement for traditional cessation services. An important caveat is that not all Australians are digitally literate or have digital access. The Australian Digital Inclusion Index (ADII) assesses digital inclusion across the dimensions of access, affordability, and digital ability [25]. Scores above 65 indicate high levels of digital inclusion, scores between 45 and 65 indicate moderate levels of digital inclusion, and scores below 45 indicate low levels of digital inclusion. The national average ADII score has improved over the past 4 years, from 52.7 in 2014 to 56.5 in 2017, mainly driven by increases in access and digital ability, with smaller improvements in affordability. However, there are clear divides across the social spectrum. In 2017, the average ADII score was 41.1 in low-income households compared with 68.1 in high-income households, 42.9 in those aged 65 years and older, and 49.5 in Indigenous Australians. Inclusion is also higher in cities (ADII score 58.6) than in rural areas (ADII score 50.7), although this gap has slightly narrowed since 2015 [25]. Importantly, these less digitally included groups also have the highest smoking rates. A survey of disadvantaged Australian smokers found similar themes; internet use was negatively associated with older age, heavier smoking, and lower income [10].

### Comparison With Prior Work

Our study thus provides important new insights into a hitherto unstudied group. To date, all intervention trials of smoking cessation mobile health (mHealth) apps have targeted younger (<50 years old) populations [8]. By contrast, the mean age of our population was 62. All participants appeared confident with IT use, as they reported often using online banking and, less frequently, online shopping, and they had higher-than-average education. Although most invitees did not respond and did not provide a reason to decline enrollment, we did uncover evidence of the digital divide; of the 40 invitees who were ineligible, 13 (33%) did not own a smartphone or a computer. This figure is similar to that reported in the study by McCrabb et al [10], in which 28% of socially disadvantaged smokers did not have any internet access. We did not target Indigenous Australians in this study; besides, only (1/64, 2%) of our participants self-identified as Indigenous Australian, and we therefore cannot comment on app acceptability in this group. A pilot study that evaluated the use of a smoking cessation app in Indigenous Australians, which was limited by a small sample size, found low use of the trial app; however, participants valued social media interaction and distraction elements, such as games in apps [26]. The My QuitBuddy app contains these features, and may thus prove acceptable to smokers among Indigenous Australians. Future work with Indigenous Australians is much needed as smoking prevalence in this group is 2.8 times more than that of non-Indigenous Australians, and is responsible for roughly twice the disease burden (17%) [27].

### Limitations

This pilot study examined the feasibility of running a larger statistically powered randomized controlled trial. The favorable trends toward better quit outcomes support the need for a larger trial. An important factor we observed was that only about one-quarter of invited individuals consented to be randomized. A large proportion of people did not respond to the invitation. Although perhaps one-third of nonrespondents may be affected by digital access/literacy issues, we believe a major limitation could have been our hospital telephone systems, which do not display caller ID, thus increasing the chance of the researcher’s call being blocked. In the future, we would prioritize SMS text messages and email invitations over phone calls. Nevertheless, once recruited, participants seemed motivated and the 91% (58/64) retention rate is acceptable. Another limitation often discussed in smoking cessation trials is biochemical verification of smoking status self-reported by participants. We did not feel that a hospital visit specifically for biochemical verification was practical. Attempts at remote biochemical testing have been made with limited success. For example, return-of-post cotinine tests and personal exhaled carbon monoxide monitors, evaluated in pilot studies, led to disappointing (25%-50%) returns and would probably be impractical in large, pragmatic studies of mHealth apps [28,29].

### Future Work

A unique difference that mHealth apps offer, which traditional smoking cessation interventions are unable to realistically deliver, is the proximity and longevity of smoking cessation support. Apps remain on the smartphone until deletion, potentially providing daily motivational support for years. This is important because nicotine addiction is a chronic relapsing-remitting disease. Pharmacotherapy and counseling are generally used for a finite period and show a significant loss of effect over time. It is conceivable that once a smoker quits, abstinence is better maintained when using an app. A 12-month follow-up period may be able to test this hypothesis.

Future studies could evaluate the effectiveness of individual app components for smoking cessation. Participant surveys only scratch the surface of this question. Passive collection of real-time “backend” data will give insights into how and when users interact with the app, how this varies by demographics, and how this may change over time. Future trials, with suitable data protection and ethical consideration, should capture these important data to inform improvements in app design.

### Conclusions

mHealth apps are an emerging technology that hold great promise for behavior change as an adjunct to standard cessation services. Apps may have a role in older smokers, but the evidence base is weak and needs urgent attention. Our pilot study suggests that apps are acceptable to a sizeable minority of older smokers, but that they may improve smoking cessation outcomes in those who engage with them. As older generations become increasingly IT literate, and if digital equality can be improved, it is possible that the acceptability of apps will increase. The encouraging findings from this pilot study remain to be tested in a larger, statistically powered trial.

## Appendix

Multimedia Appendix 1 CONSORT-eHEALTH checklist (V1.6.1).

## Abbreviations

ADII: Australian Digital Inclusion Index
ILST: International Lung Screen Trial
IT: information technology
uMARS: User Version of the Mobile App Rating Scale
